# Acoustic omni meta-atom for decoupled access to all octants of a wave parameter space

**DOI:** 10.1038/ncomms13012

**Published:** 2016-09-30

**Authors:** Sukmo Koo, Choonlae Cho, Jun-ho Jeong, Namkyoo Park

**Affiliations:** 1 Photonic Systems Laboratory, Department of Electrical and Computer Engineering, Seoul National University, Seoul 08826, Korea; 2 Department of Nano Manufacturing Technology, Korea Institute of Machinery and Materials, Daejeon 34103, Korea

## Abstract

The common behaviour of a wave is determined by wave parameters of its medium, which are generally associated with the characteristic oscillations of its corresponding elementary particles. In the context of metamaterials, the decoupled excitation of these fundamental oscillations would provide an ideal platform for top–down and reconfigurable access to the entire constitutive parameter space; however, this has remained as a conceivable problem that must be accomplished, after being pointed out by Pendry. Here by focusing on acoustic metamaterials, we achieve the decoupling of density *ρ*, modulus *B*^−1^ and bianisotropy *ξ*, by separating the paths of particle momentum to conform to the characteristic oscillations of each macroscopic wave parameter. Independent access to all octants of wave parameter space (*ρ*, *B*^−1^, *ξ*)=(+/−,+/−,+/−) is thus realized using a single platform that we call an omni meta-atom; as a building block that achieves top–down access to the target properties of metamaterials.

## Abstract (web summary)

For meta-atoms, achieving full access to the wave parameter space remains a problem. Here, Koo *et al*. outline the criteria for the decoupling of wave parameter from first principles, and realize an omni meta-atom that achieves independent, broad-range access to all octants of acoustic parameter space.

## Graphical abstract

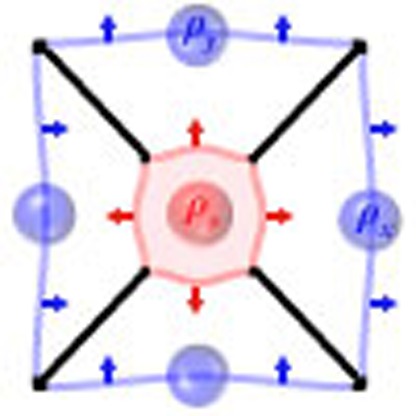

The general features of wave propagation are ultimately determined by the properties of its medium, where the wave travels through. In order to achieve an extreme manipulation of wave propagation, the accessibility to the unusual space of wave parameters is, therefore, obligatory. A wide variety of extreme wave parameters and their applications have been realized for different waves and material systems in the context of metamaterials; spanning the fields of, acoustics^1^,^2^,^3^,^4^,^5^,^6^,^7^,^8^,^9^,^10^,^11^,^12^,^13^,^14^,^15^, photonics^16^,^17^,^18^,^19^,^20^,^21^,^22^,^23^,^24^,^25^,^26^,^27^,^28^,^29^,^30^,^31^,^32^,^33^,^34^,^35^,^36^,^37^, thermodynamics^38^, elasto-dynamics^39^,^40^,^41^, seismics^42^, among others. Negative-1,2,3,4,16,17,18,39, zero-5,6,7,19,20,21, ultrahigh-index^22^, hyperbolic-23, anisotropic-8,9,24, bianisotropic-24,25,26, chiral-16,24 and disordered-metamaterials^27^ have been demonstrated, along with their applications towards cloaking^19^,^28^,^29^, super-focusing^8^, perfect absorption^10^, iso-spectrality^27^, meta-surface hologram^30^ and frequency-agile memory^31^.

With keen interest on applications, reconfigurable control of wave parameters has also become main stream in wave physics^28^,^32^,^33^. Nonetheless, although the decoupling of fundamental wave parameters has been envisaged as an ideal platform towards the top–down and deterministic reconfiguration of the meta-atom (Pendry *et al*.^28^), its feasibility has remained merely as a plausible idea that has yet to be proven. In most cases, the decoupling of constituent parameters has been achieved via the combination of elementary resonators in a non-isotropic and polarization-dependent form^17^,^18^,^29^,^39^. At present, strategies for metamaterial design have been based on bottom–up approaches; in which building blocks are proposed first, and subsequent design is performed iteratively until a specific index and impedance are achieved. Although it has been recently reported that pentamode metamaterials can provide decoupled access to all positive mechanical wave parameters^40^, achieving full accessibility to the entire space of wave parameters with the designs of existing metamaterials remains an open question, and the existence of an omnipotent meta-atom platform for reconfigurable and seamless access to the wave parameter space also has yet to be answered.

Inspired by the fundamental oscillations of the elementary particle of a wave, in this communication we propose an entirely new design strategy for the meta-atom. Focusing on an acoustic platform, the criteria for the decoupling of wave parameters are derived from first principles, and an omni meta-atom that achieves independent, broad-range access to all octants of the wave parameter space (*ρ*, *B*^−1^, *ξ*) is demonstrated. Based on the top–down access capability for target (*ρ*, *B*^−1^, *ξ*), we then demonstrate a new class of meta-devices; bianisotropic meta-surfaces for independent beam shaping of transmission- and reflection-waves, and as well as zero-index waveguides for pressure–velocity conversion. Our work provides a deeper insight on the relationship between wave parameters and the internal structures of a meta-atom, and paves a new route towards systematic access to target wave parameters.

## Results

### First-principle derivations of effective wave parameters

**Figure 1:**
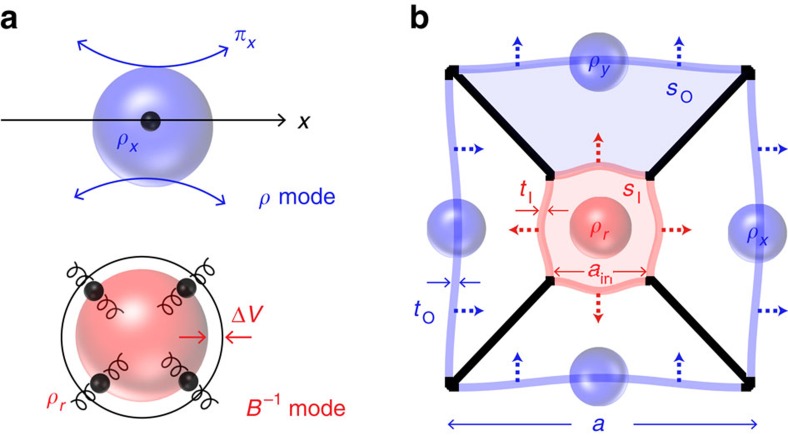
Characteristic oscillations of acoustic atoms and the decoupling of constitutive wave parameters. (**a**) Linear and radial characteristic oscillations of acoustic atoms for *ρ* and *B*^−1^, respectively. (**b**) Schematic of the proposed meta-atom. blue and red: outer and inner membranes; black: solid wall; *t*_O(I)_: outer (inner) membrane thickness; *s*_O_ (shaded in light blue) and *s*_I_ (shaded in light red): outer and inner area (region) in the meta-atom unit cell.

Understanding that the electromagnetic wave parameters *ɛ* and *μ* of a classical atom are directly related to the linear- and angular-oscillations of an electron respectively, the insight of this study begins from the characteristic oscillation of elementary particles, in relation to wave parameters of interest. In this respect, the derivation of effective parameters for an acoustic wave (*ρ*_*x*_: density for *x* direction, *B*: bulk modulus) from the characteristic motions of acoustic particles is straightforward (Fig. 1a).

Based on the duality between electromagnetic and acoustic waves^5^,^11^, we first modify Alù's derivation of electromagnetic macroscopic wave parameters^34^, to derive effective parameters of an acoustic system from first principles (details in Supplementary Note 1). In the limit of a long wavelength (|**β**|*a*<<*π*, **β**: effective wavevector, *a*: lattice constant), *B*^−1^ and *ρ*_*x*_ are then expressed as,


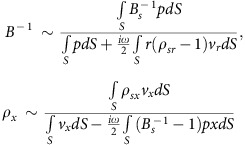


for a two-dimensional unit cell *S*; with a distributed particle density tensor *ρ*_*s*_ (subscripts *x*, *r* denote density directions) and modulus *B*_*s*_ (normalized to air) of the constituting materials inside *S*, where **r** is the position vector measured from the cell centre, and *p* and **v** each correspond to the pressure and velocity fields at **r**.

### Design criteria for the decoupling of wave parameters

Important to note from equation (1) is the presence of cross-coupling terms in the denominators of *B*^−1^(*ρ*_*sr*_) and *ρ*_*x*_ (*B*_*s*_^−1^), which hinder the decoupled access to *B*^−1^ and *ρ*_*x*_. Out of various possibilities, we try to spatially decouple *B*^−1^ and *ρ*_*x*_, by employing a meta-atom with an inner sub-cell (IS) of radial symmetry and outer sub-cells (OS) of decoupled *x*, *y* linear vibrations, each conforming to the fundamental oscillations of *B*^−1^ and *ρ*_*x,y*_ (Fig. 1a); in a square lattice composed of a membrane, air and solid walls (Fig. 1b).

Under these settings, the expansion near the Dirac point^5^,^6^,^19^ of zero compressibility and zero density constrain the movement of outer- and inner-membranes, enabling further reduction of the equations; radial movement of the outer membrane is prohibited (as *B*^−1^∼0), and outer- and inner-membrane should move out of phase but with the same momentum value (as *ρ*_*x*_∼0). In this case, by employing a heavy mass for *ρ*_m_ (or a large thickness for the inner membrane), equation (1) is reduced to (details in Supplementary Note 2),


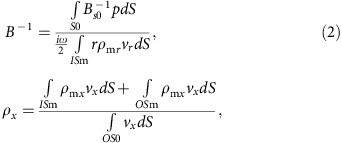


where subscript m and 0 denote the material (membrane and air) for the given physical quantities (for example, *ρ*, *B*^−1^) at *S*, IS and OS. Most importantly, equation (2) shows the direct control of effective *B*^−1^ with mass *ρ*_m_ of the inner-membrane in the denominator, which justifies the proposed approach of dividing the meta-atom into the inner- and outer-sub-cells that correspond to the fundamental oscillations of *B*^−1^ and *ρ*_*x*_. With the inner membrane mass *ρ*_m_ determined for *B*^−1^∼0, then the control of effective *ρ*_*x*_ with the tuning of only outer-membrane mass (that is, second term *ρ*_m*x*_ in the numerator of *ρ*_*x*_) is consequently realized.

A more explicit solution for the structure shown in Fig. 1b can be obtained by using the coupled mode theory (CMT). Applying Newton's law to the membranes and Hooke's law to the air region, the decoupled relation for *ρ* (proportional to outer membrane mass *ρ*_Al_*t*_O_) and *B*^−1^ (proportional to inner membrane mass *ρ*_Al_*t*_I_) are again confirmed in the long-wavelength limit, as shown in equation (3) (derivation in Supplementary Note 3, see also Supplementary Fig. 1):


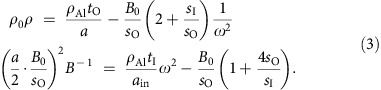


where *ω* being the angular frequency, *ρ*_0_ and *ρ*_Al_ being the density of air and Al membrane, *a* being the lattice constant, *a*_in_ being the size of the IS, *t* being the membrane thickness and *s* denotes the area of inner- (I) and outer- (O) sub-cells, respectively. Worth to mention, with equation (3), it is also possible to achieve independent control of (*ρ*, *B*^−1^) as a function of pressures (∼*B*_0_) or volumes/areas (∼*s*_O_, *s*_I_) in sub-cells.

### Demonstration of the decoupling of effective parameters

**Figure 2:**
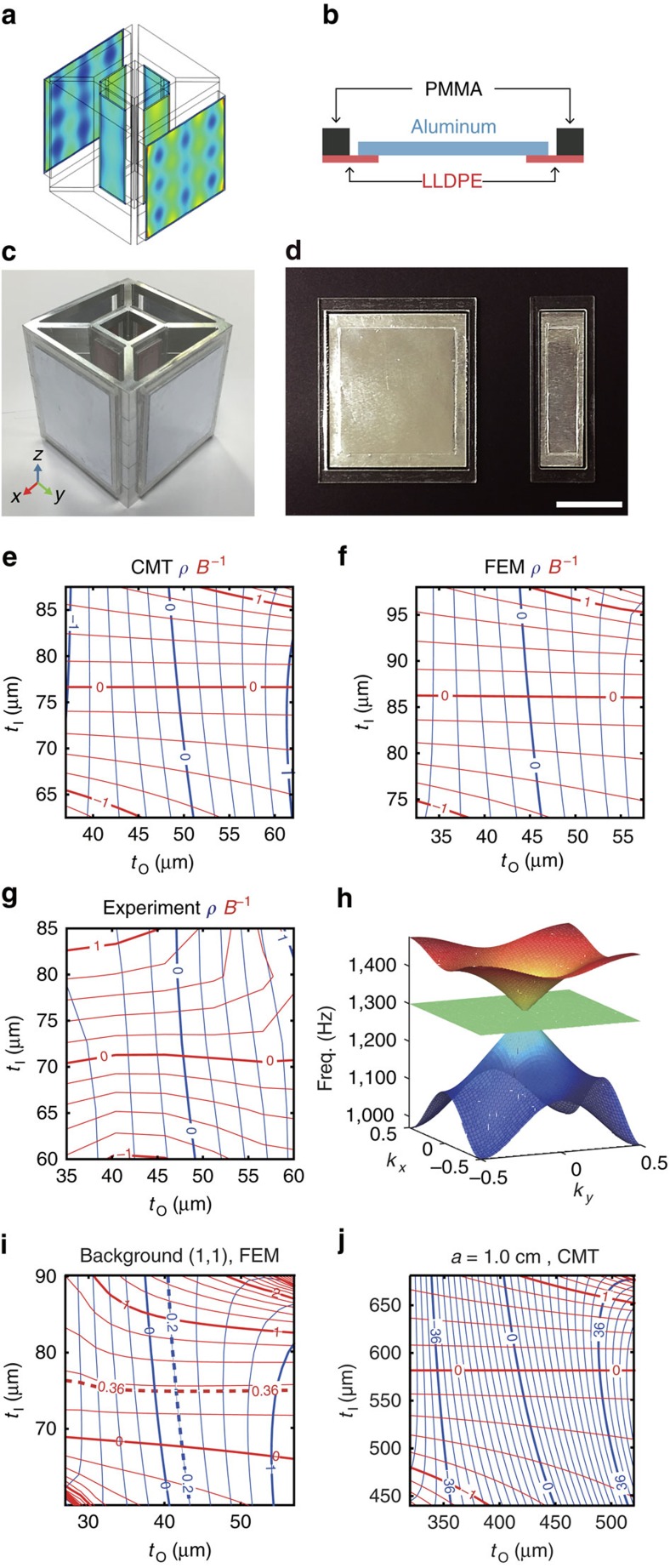
Experimental realization of the meta-atom and the decoupling of effective wave parameters. (**a**) Deflection pattern of the membrane obtained from three-dimensional FEM. (**b**) Structure of the membrane. (**c**,**d**) Three-dimensional experimental realization of the meta-atom and membrane (white line denote scale bar, 20 mm). (**e**) CMT (**f**) two-dimensional FEM and (**g**) experimental results showing decoupled access to *ρ* (blue lines) and *B*^−1^ (red lines) with outer and inner membranes thicknesses *t*_O_ and *t*_I_, respectively. Except (**j**) grid spacings for *ρ* (blue lines) and *B*^−1^ (red lines) are 0.2. The thickness of the Al wall in the FEM and experiment is set at 3 mm. The lattice constant and height of the cell were 6 and 7 cm, respectively. In the experiment, measurements were taken with a membrane thickness resolution at 10 μm. (**h**) CMT obtained dispersion relation of the meta-atom, with the design parameter of (*t*_O_=49.3 μm, *t*_I_=76.6 μm). (**i**) Shifting the centre of the decoupling operation to (*ρ*_c_, *B*_c_^−1^)=(0.2, 0.36) (intersection of the thick dashed lines), with the effective medium approach (placing the meta-atom in a host medium of air. see Supplementary Fig. 4 and Supplementary Note 5). (**j**) Extension of density tuning range (−36<*ρ*<36) with the use of smaller unit cell (*a*=1 cm) having heavier mass plates (see Supplementary Fig. 5 and Supplementary Note 5). Grid spacings for *ρ* and *B*^−1^ for **j** are 3 and 0.2. Operation frequency is 1,300 Hz, for all cases.

The meta-atom's membrane motion calculated by the finite element method (FEM using commercially available program COMSOL), and the schematic of the membrane are shown in Fig. 2a,b, respectively. Experimental realizations of the meta-atom and membranes are shown in Fig. 2c,d. Details of the structure and material parameters are described in the Methods section. Using the un-approximated CMT solutions in Supplementary Eq. (14), in Fig. 2e we visualize the mapping of (*ρ*, *B*^−1^) in terms of membrane thickness of outer- and inner-sub-cell (*t*_O_, *t*_I_) at 1,300 Hz, near the Dirac point^5^,^6^,^19^ (Fig. 2h and Supplementary Fig. 2 in Supplementary Note 3). From Fig. 2e, perfectly orthogonal decoupling between *ρ* and *B*^−1^ is evident, especially near (*ρ*, *B*^−1^)=(0, 0), which is in excellent agreement with FEM and experimental results (Fig. 2f,g). The membrane movements of (*ρ*, *B*^−1^) modes exhibit dipolar- and monopolar- patterns, confirming the proposed ansatzs and discussions regarding Fig. 1 and equation (2) (see Supplementary Note 4 and Supplementary Fig. 3). It is important to note that, inverse determination of the meta-atom structural parameter (*t*_O_, *t*_I_) is also possible from the target (*ρ*, *B*^−1^) values using equation (3) or Fig. 2e. Finally, we also show decoupled operation away from the Dirac point (Fig. 2i) and extension of the tuning range (Fig. 2j), with the use of effective medium approach and smaller unit cell of heavier membranes, respectively (Supplementary Note 5 and Supplementary Figs 4 and 5).

### Decoupled implementation of acoustic bianisotropy

**Figure 3:**
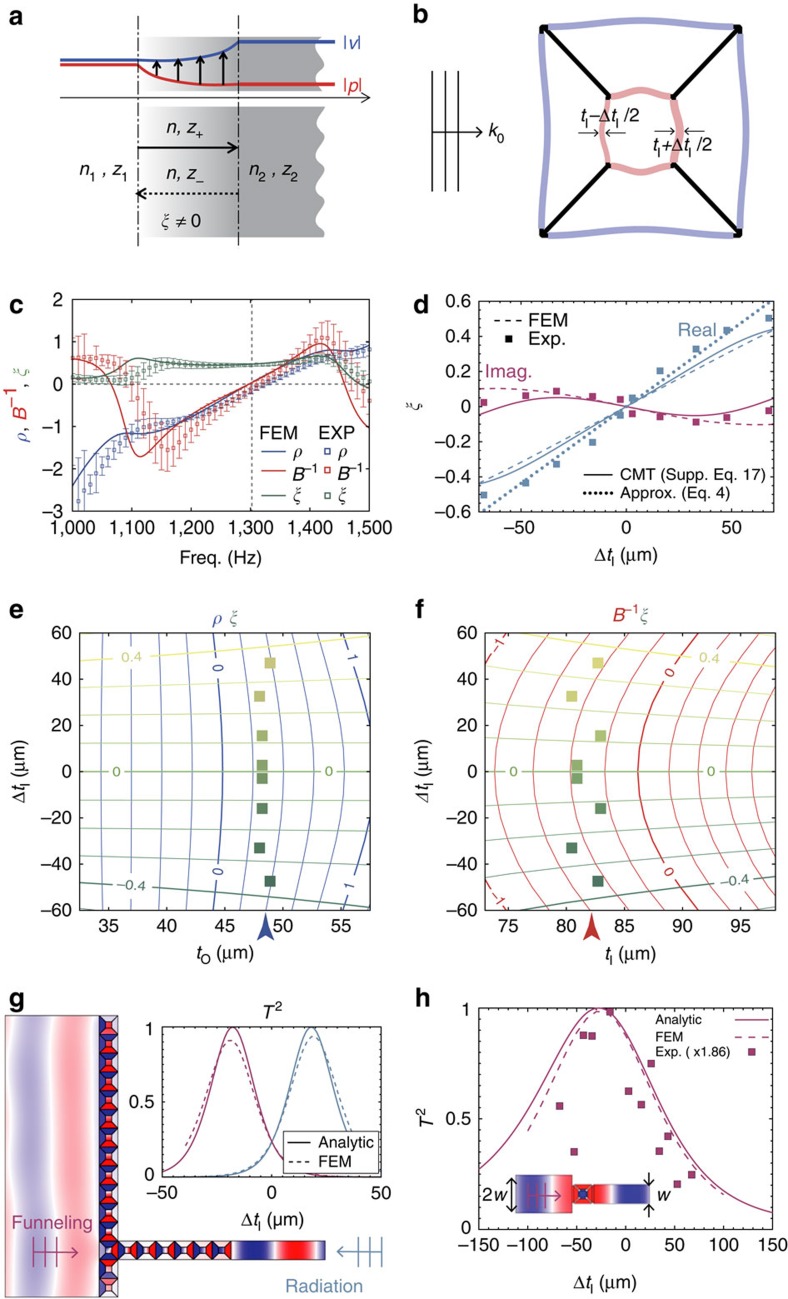
Implementation of acoustic bianisotropy and bianisotropic meta-waveguide. (**a**) Exchange of velocity- and pressure-field amplitude (kinetic and potential energies) in an acoustic bianisotropic medium. (**b**) Schematic of the bianisotropic meta-atom with an asymmetric arrangement of the membrane thickness. (**c**) FEM calculated, and experimentally measured wave-parameter spectra of the bianisotropic meta-atom, designed to achieve (*ρ*, *B*^−1^, *ξ*)=(0, 0, 0.5) at 1,300 Hz. The zero density and zero compressibility at the operation frequency of 1,300 Hz are the result of zero crossing between two nearby resonances (∼1,100 and 1,400 Hz). (**d**) Tuning of *ξ* with Δ*t*_I_ from the experiment (square symbols), CMT (solid line), FEM (dotted lines), and the approximation (point symbols, equation (4)). (**e**,**f**) Tuning of (*ρ*, *ξ*) and (*B*^−1^, *ξ*) with Δ*t*_I_. (**g**) FEM-calculated pressure field for the super-funnelling (*w*_1_/*w*_2_=15, *ξ*=−0.158, Δ*t*_I_=−18 μm). The inset shows the transmittance for super-funnelling and super-radiation. (**h**) Experimentally obtained super-funnelling with a single meta-atom at different Δ*t*_I_ (or bianisotropy *ξ*). *w*_1_/*w*_2_=2. Operation frequency is 1,300 Hz, for all cases.

The decoupling could be generalized to include the other wave parameter axis of bianisotropy^24^,^25^,^26^. Meanwhile the bianisotropy *ξ* has been demonstrated using Ω-type metamaterials^25^,^26^ in nano-photonics, yet it needs to be conceptualized and demonstrated for acoustic metamaterials. In parallel to electromagnetic bianisotropy that exchanges kinetic and potential energies (or equivalently, electric and magnetic fields), here we investigate the coupling constant *ξ* that connects the velocity and the pressure field (Fig. 3a). Considering that *ξ* is related to structural asymmetry^34^, we choose to apply asymmetry in the form of *t*_I_±Δ*t*_I_/2, in order to create non-zero *ξ* (Fig. 3b,c). The analytically derived *ξ* (Supplementary Eq. (17) in Supplementary Note 6; its approximation in equation (4)) shows a linear relation with Δ*t*_I_, independently from *t*_I_ or *t*_O_ (Fig. 3d and Supplementary Fig. 6). Therefore, near-perfect decoupling between *ρ*, *B*^−1^ and *ξ* near (0, 0, 0) (Fig. 3e,f) is realized, in excellent agreement with the numerical and experimental results.


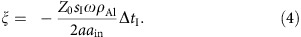


### Asymmetric impedance manipulation based on the bianisotropy

The salient feature of the bianisotropic medium is in the asymmetric impedance manipulation of the wave with the exchange in kinetic and potential energy during wave propagation. Using the bianisotropic meta-atom at a matched zero index, here we report a perfect transmission between over-*λ* and sub-*λ* widths (or impedances) of waveguides (Fig. 3g); achieving super-funnelling/radiation above the *λ*-zone limit^43^,^44^.

As shown in Fig. 3g, six bianisotropic zero index meta-atoms (*ξ*=−0.158 and *ρ*=*B*^−1^=0) in the output waveguide, in addition to a layer of meta-atoms of (*ρ*, *B*^−1^, *ξ*)=(0, 0, 0) in the input side are used. The non-zero *ξ*=−0.158 value for ideal exponential field evolution and complete impedance conversion is calculated from the ratio of input/output waveguide widths and the number of bianisotropic meta-atoms (*ξ*=log(*w*_1_/*w*_2_)/(2*k*_0_*˙*6*a*); see Supplementary Note 7 and Supplementary Fig. 7). Achieving exact (*ρ*, *B*^−1^, *ξ*) values for the meta-atoms from the independent control of (*t*_O_, *t*_I_, Δ*t*_I_), Fig. 3g shows the pressure field calculated by the FEM, exhibiting the super-funnelling from over-*λ* to the sub-*λ* width waveguide (*w*_1_/*λ*=3.4 to *w*_2_/*λ*=0.227, *w*_1_/*w*_2_=15). Reflectionless, ideal impedance conversion with the bianisotropic zero-index waveguide is clear; in addition to the suppression of higher order mode excitation from the meta-atom array of matched zero index. Excellent agreement with analytical result (Supplementary Eq. (24) in Supplementary Note 8) for an extreme case of a single meta-atom for (*w*_1_/*w*_2_)=2 are also demonstrated experimentally (Fig. 3h).

### Bianisotropic meta-surface for decoupled T and R wavefront

As a final application example supported by the capability of independently addressing target *ρ*, *B*^−1^, and also *ξ* values, we demonstrate a bianisotropic wave front shaping in a meta-surface^12^,^13^,^30^,^35^,^36^,^37^ context, of critical novelty in a transmission-reflection (T and R) decoupled form. Under the notion of the generalized Snell's law^35^, we emphasize that the transmission- and reflection-decoupled bianisotropic wave front shaping can be achieved only via independent control of (*ϕ*_R_, *ϕ*_T_) at the meta-surface; where the controllability of *ξ* for individual meta-atom has a critical role in achieving *n*_R_≠*n*_T_ while maintaining the same value of *n*_eff_ over the entire surface.

**Figure 4:**
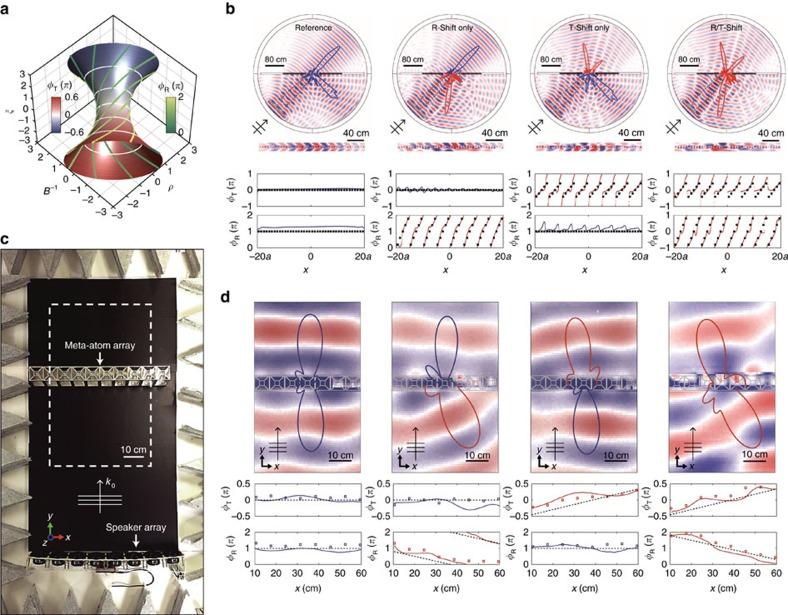
Transmission and reflection decoupled wave front shaping using a bianisotropic meta-surface. (**a**) Phase shift contour (*ϕ*_R_, *ϕ*_T_) in the parameter octant space of (*ρ*, *B*^−1^, *ξ*) for a 50:50 power division for the transmitted and reflected waves (Supplementary Note 9). (**b**) FEM calculated pressure field patterns for an incidence wave from the bottom at 45°. Left to right: reference, shift in the reflection, transmission, and reflection and transmission wavefront. The overlaid far-field polar plots are calculated from the near-field data. Transmission and reflection phases of the meta-atom array (dotted lines are for the design; the solid lines are measured from the FEM calculations) are shown at the bottom of each figure. (**c**) Top view of the experimental set-up. The experiment was performed with a 10 × 1 meta-atom array in a 70 × 150 × 7 cm box with an acoustic absorber and an 8 × 1 speaker array. (**d**) Experimentally measured scattered pressure field patterns and calculated far-field polar plots for a normal incidence wave from the bottom. Below the field patterns, the transmission- and reflection-phases of the meta-atom array are shown. Dotted lines for design, square marks from the one-dimensional impedance tube measurements, and solid lines from the experimentally measured pressure field near the meta-surface. Operation frequency is 1,300 Hz, for all cases.

To realize independent controllability of (*ϕ*_R_, *ϕ*_T_), in Fig. 4a we plot the phase shift contour (*ϕ*_R_, *ϕ*_T_) in the parameter octant space of (*ρ*, *B*^−1^, *ξ*), achieving 50:50 power division for the transmitted and reflected waves (details in Supplementary Note 9 and Supplementary Fig. 8). It is stressed that, in the absence of bianisotropy (*ξ*=0), it is impossible to adjust (*ϕ*_R_, *ϕ*_T_) under the given 50:50 power splitting condition, as evident from Fig. 4a. From target phase shifts (*ϕ*_R_, *ϕ*_T_) of an individual meta-atom (in a 40 × 1 array), calculations of (*ρ*, *B*^−1^, *ξ*) are obtained from Supplementary Eq. (25) (details in Supplementary Note 9 and Supplementary Fig. 9), which achieves ordinary (Δ*ϕ*(*x*)=0) or anomalous (Δ*ϕ*(*x*)≠0) transmission and reflections (Supplementary Fig. 10). Subsequent, top–down determination of the corresponding (*t*_O_, *t*_I_, Δ*t*_I_) from (*ρ*, *B*^−1^, *ξ*) is then straightforward. Independent control of the reflected wave, the transmitted wave, as well as the simultaneous control of the reflected- and transmitted-wave compared with the reference (left figure), with corresponding phase maps at the bottom of the figures are shown in Fig. 4b.

Experimental realization of a bianisotropic meta-surface has also been carried out using a 10 × 1 meta-atom array, in a 70 × 150 × 7 cm box with an acoustic absorber and an 8 × 1 speaker array (Fig. 4c). With the finite dimension of the set-up used in this study, experiment has been performed with an incidence wave normal to the meta-atom array. Figure 4d shows scattered pressure field patterns together with the reflection- and transmission-phase (*ϕ*_R_, *ϕ*_T_) of individual meta-atoms; dotted lines from the target design, square marks from the impedance tube measurements, and solid lines from the pressure field scanning measurements. With precise access to (*ϕ*_R_, *ϕ*_T_) values from the control of (*ρ*, *B*^−1^, *ξ*) in each meta-atom, decoupled manipulation of the reflection- and transmission-wave fronts are successfully realized experimentally.

## Discussion

In summary, with the insight gained from fundamental oscillations of the wave supported by first principles of homogenization theory, we demonstrated an acoustic omni meta-atom that achieves decoupled access to the target wave-parameter in the octant space of (*ρ*, *B*^−1^, *ξ*), with the tuning of structural factors of the meta-atom (*t*_O_, *t*_I_, Δ*t*_I_). Excellent agreements between CMT-based solutions, FEM-based numerical analysis and experiments have been realized, confirming the top–down design capability that addresses target (*ρ*, *B*^−1^, *ξ*) values. Shifting the centre of decoupling operation in the frame of effective medium theory, as well as the extension of effective parameter tuning range have been verified numerically, in agreement with the proposed theory. The feasibility of active tunability using pressures and volumes in sub-cells has also been discussed with CMT equations. With the capability of independently- and deterministically-addressing target wave parameters, we show applications of bianisotropic pressure–velocity impedance conversion, and reflection-transmission decoupled wave front shaping. We note that, likewise most of resonance based metamaterials, the bandwidth limitation in our design is considered as an engineering subject which could be mitigated with various approaches^3^,^10^,^23^. Our work opens a new paradigm in the design of meta-atoms by overcoming difficulties observed from the bottom–up approach, and provides an ideal platform by resolving the previously envisaged but unanswered issue of the decoupled excitation of constitutive parameters. Using the same approach, we expect further extension of decoupled access to other waves (that is, ultra-sonic^45^, electromagnetic, elastic^39^ and thermal) and wave parameters (that is, stress, strain, gyrotropy and chirality).

## Methods

### Details of the meta-atom structure and material parameters

For the experiment, we constructed a two-dimensional slab meta-atom (height=7 cm) using an Al sheet-loaded linear low-density polyethylene (LLDPE) membrane and a solid Al wall (thickness=3 mm, *a*=6 cm, *a*_in_=2 cm; see Fig. 1a). To achieve the decoupling of (*ρ*, *B*^−1^) at 1,300 Hz, the effective thicknesses (*t*_O_, *t*_I_) of the Al sheet have been controlled between 35–60 and 50–90 μm, respectively; same thicknesses used in CMT and FEM analyses. The densities^14^,^46^ of air and Al are assumed to be 1.21 and 2,700 kg m^−3^. The wave parameters of interest have been calculated by using *S* parameters extracted from a 4-point measurement impedance tube. It is noted that we employed a composite membrane constructed with an Al-sheet mounted on top of a larger frame of an LLDPE film as shown in Fig. 2b. The Al-sheet has a much greater weight and stiffness compared with the LLDPE film, and provides a method of controlling the composite membrane mass with its thickness; independently of the stiffness of the composite membrane (*k*^−1^_composite_∼*k*^−1^_LLDPE_+*k*^−1^_Al_) that is primarily determined by the properties of the LLDPE film (10 μm thick). For the fine tuning of the membrane's effective thickness (that is, mass), we used 3–8 stacked layers of Al-sheets that each had a thickness of ∼15 μm with periodically perforated disks of a 2-mm radius. The final dimensions of the outer (inner) LLDPE film and the Al-sheet were 54 × 60 mm (18 × 60 mm) and 52 × 58 mm (16 × 58 mm), respectively.

### Data availability

All relevant data that support the findings of this study are available from the corresponding author upon request.

## Additional information

**How to cite this article:** Koo, S. *et al*. Acoustic omni meta-atom for decoupled access to all octants of a wave parameter space. *Nat. Commun.* **7**, 13012 doi: 10.1038/ncomms13012 (2016).

## Supplementary Material

Supplementary Information Supplementary Figures 1-10, Supplementary Notes 1-9 and Supplementary References.
