# Supplementary material for: Acoustic omni meta-atom for decoupled access to all octants of a wave parameter space
Source: Nat Commun. 2016 Sep 30;7:13012. doi: 10.1038/ncomms13012 (PMC5056457; doi:10.1038/ncomms13012)
Supplement: Supplementary Information — Supplementary Figures 1-10, Supplementary Notes 1-9 and Supplementary References. [file ncomms13012-s1.pdf]

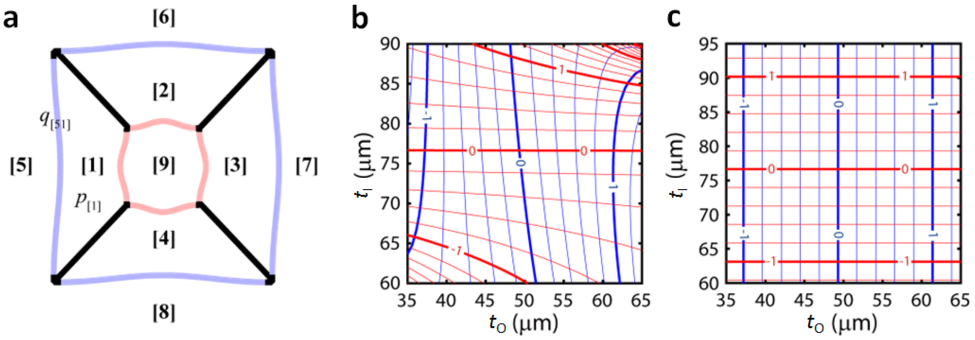

Supplementary Figure 1 | Schematics of the meta-atom and decoupled control of wave parameters. a, Assignment of average pressure $p_{[i]}$ and displacement $q_{[jk]}$ for the membranes and sub-cells. **b**,**c**, Mapping of (ρ, B^{-1}) with the control of (t_{O}, t_{I}) using **b**, the full solutions in (14) and **c**, their approximations (15) (right) at 1,300 Hz.

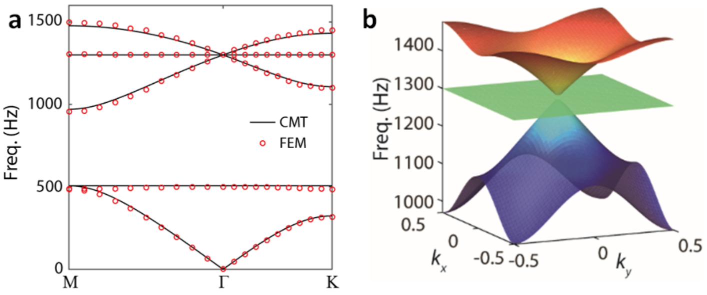

Supplementary Figure 2 | Band structures of the meta-atom. a, 2D band diagram (CMT and FEM) and **b**, 3D plot of Dirac cone (CMT) near the operation frequency at 1,300 Hz.

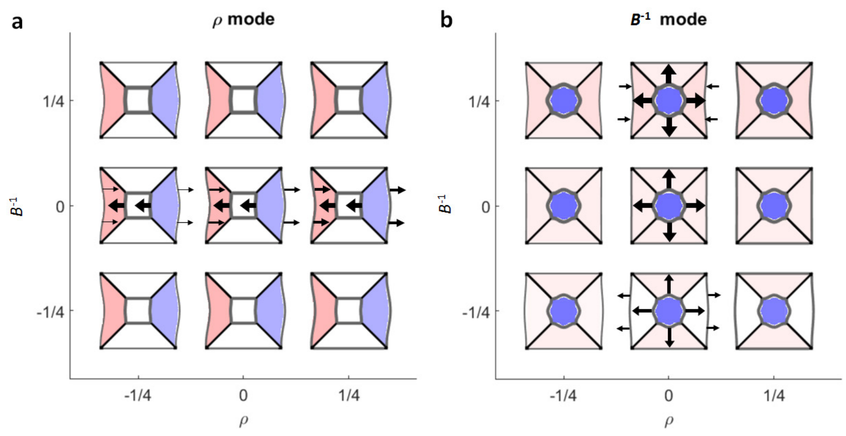

Supplementary Figure 3 | Characteristic motions of the meta-atom for **a**, ρ-dipolar and **b**, B^{-1}-monopolar modes. Displacement (gray lines) and momentum (black arrows) of the membranes. Also shown are the pressure field patterns (+: red, -: blue) at 1,300 Hz.

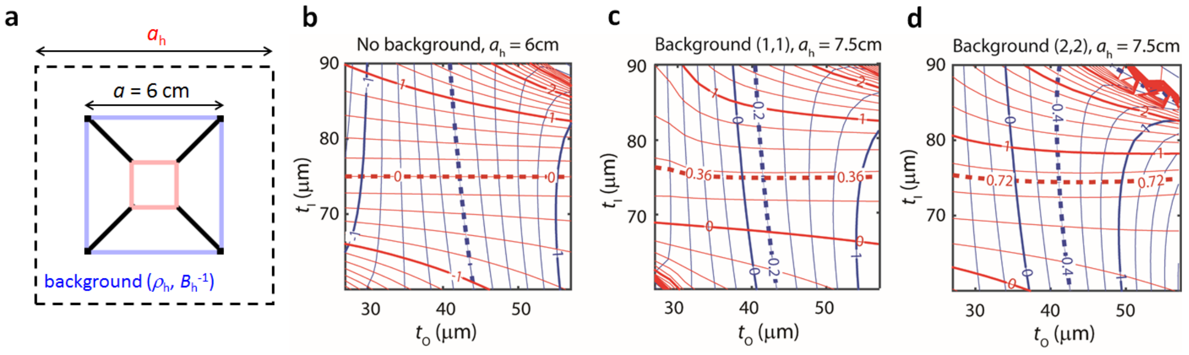

Supplementary Figure 4 | Shifting the center of decoupling operation away from the Dirac point. a, Schematics of the meta-atom in the background host medium. FEM obtained (ρ, B^{-1}) tuning map at 1,300 Hz are shown in **b**, (ρ_c, B_c^{-1}) = (0,0) without host ($a_h = a$) medium. **c**, (ρ_c, B_c^{-1}) = (0.2, 0.36) with (ρ_h, B_h^{-1}) = (1, 1). **d**, (ρ_c, B_c^{-1}) = (0.4, 0.72) with (ρ_h, B_h^{-1}) = (2, 2). a_h = 6cm, 7.5cm and 7.5cm for **b**, **c**, and **d** respectively.

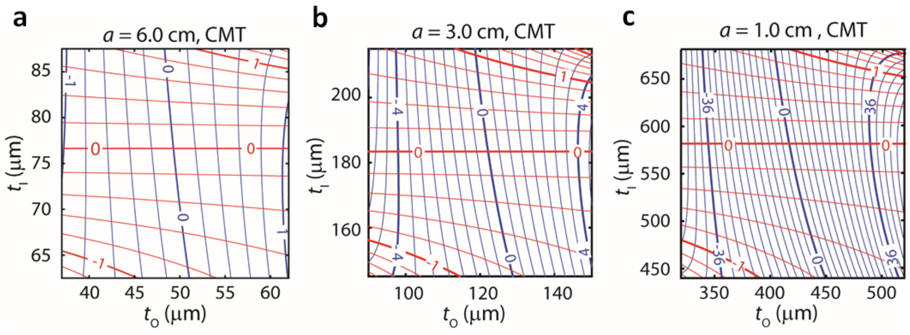

Supplementary Figure 5 | Extension of ρ tuning range. CMT calculated (ρ, B^{-1}) tuning maps at 1,300 Hz for different lattice constant, **a**, a = 6 cm, **b**, a = 3 cm and **c**, a = 1 cm. Grid spacings for B^{-1} (red lines) are fixed to 0.2, and for ρ (blue lines) are 0.2, 0.5 and 3, respectively.

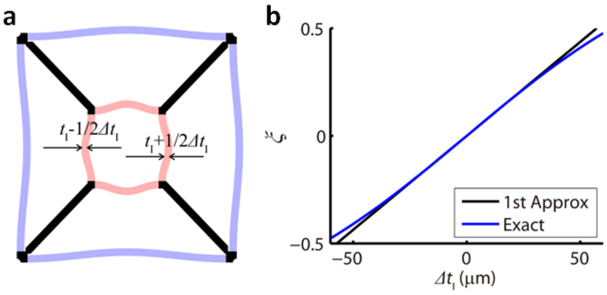

Supplementary Figure 6 | Implementation of bianisotropy and CMT obtained ξ values. a, Schematics of the asymmetric meta-atom for non-zero bianisotropy ξ with $\Delta t_\mathrm{I} \neq 0$. **b**, Comparison of exact (16) and approximated (17) solution of ξ, as a function of Δt_I. $\rho = B^{-1} = 0$, 1,300 Hz.

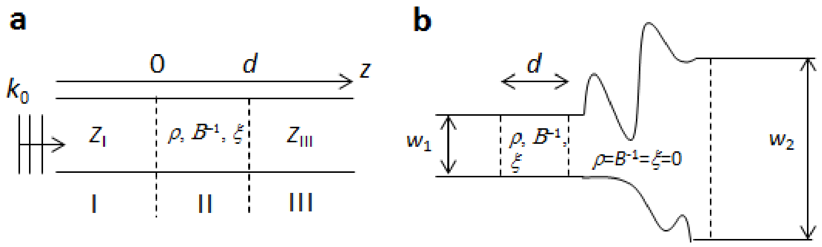

Supplementary Figure 7 | Schematics of the asymmetric transmission between **a**, different impedances or **b**, waveguides widths, using matched zero index and bianisotropic metamaterials.

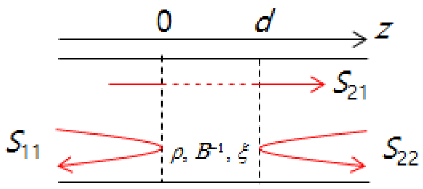

Supplementary Figure 8 | Schematics of the S parameters in the bianisotropic medium for determining effective wave parameters.

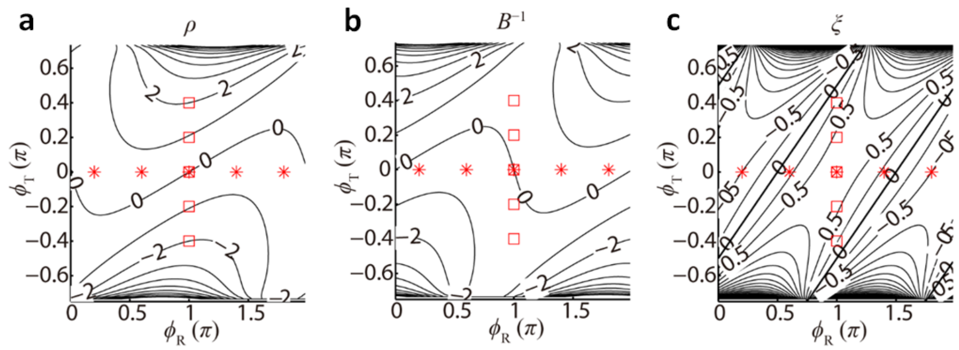

Supplementary Figure 9 | Wave parameter value maps in the (ϕ_R, ϕ_T) coordinate, subject to the constraint of 50:50 power divisions for transmission and reflection. Required **a**, density ρ and **b**, modulus B^{-1}, and **c**, bianisotropy ξ are plotted. Required phase shifts (ϕ_R, ϕ_T) for individual meta-atoms in the 40 × 1 meta-atom array, achieving extra-ordinary transmission and reflections are marked with red square ($\Delta\phi_T(x) \neq 0$) and red star ($\Delta\phi_R(x) \neq 0$), respectively (a = 6 cm, λ = 26.4 cm, and f = 1,300 Hz).

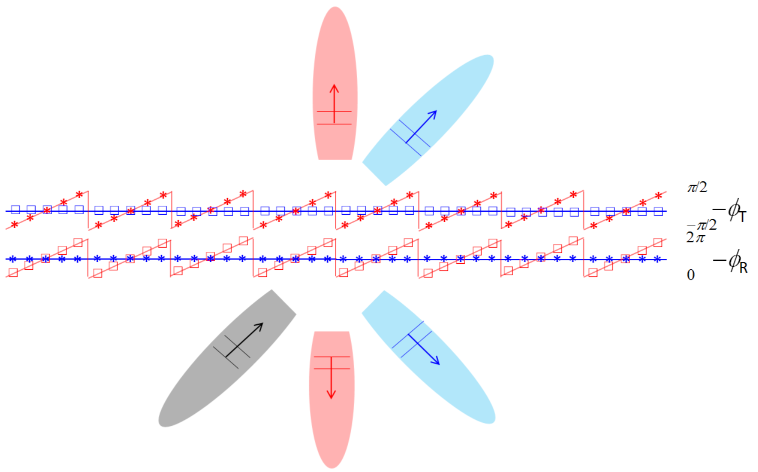

Supplementary Figure 10 | Required phase shifts (ϕ_R, ϕ_T) for individual meta-atoms in a single sheet of a 40 × 1 array to achieve ordinary (blue marks) or extra-ordinary (red marks) transmission and reflection.

Supplementary Note 1. Derivation of effective macroscopic acoustic parameters from the homogenization theory

To derive equation (1) in manuscript, we start from the microscopic acoustic wave equations[1] based on Newton's law and Hooke's law[2]. Assuming $\exp(i\omega t)$ time dependency of angular frequency ω,

$$\begin{aligned}\nabla p(\mathbf{r}) &= -i\omega\boldsymbol{\pi}(\mathbf{r}) = -i\omega\rho_0\mathbf{v}(\mathbf{r}) - i\omega\rho_0(\rho_s - 1)\mathbf{v}(\mathbf{r}) \\ \nabla\cdot\mathbf{v}(\mathbf{r}) &= -i\omega e(\mathbf{r}) = -i\omega B_0^{-1}p(\mathbf{r}) - i\omega B_0^{-1}(B_s^{-1} - 1)p(\mathbf{r})\end{aligned} \tag{1}$$

where $p(\mathbf{r})$, $\mathbf{v}(\mathbf{r})$, $e(\mathbf{r}) = B_0^{-1}B_s^{-1}p(\mathbf{r})$, $\boldsymbol{\pi}(\mathbf{r}) = \rho_0\rho_s\mathbf{v}(\mathbf{r})$ denote the pressure, velocity, strain and momentum density field at $\mathbf{r}$, respectively. ρ_0 and B_0 are the density and bulk modulus of the *air* '0'; ρ_s and B_s are the density and bulk modulus (normalized to the air) of the constituting materials in the unit cell.

By following a similar procedure used in Alù[3] (homogenization theory for electromagnetic waves), we now derive the acoustic equations for the macroscopic fields ($\overline{p}$, $\overline{\mathbf{v}}$) from (1). Noting that the effective parameters are independent of the origin of the coordinate[4,5] in case of periodic system or finite metamaterial terminated at the same location of the unit cell, we use Floquet theory with $\exp(-i\boldsymbol{\beta}\cdot\mathbf{r})$ dependence. Averaging the field of p and $\mathbf{v}$ over the two-dimensional square lattice (lattice constant a, unit cell domain S, effective wavevector $\boldsymbol{\beta}$), equation (1) then becomes

$$\begin{aligned}&-i\boldsymbol{\beta}\overline{p} = -i\omega\rho_0\overline{\mathbf{v}} - \frac{i\omega\rho_0}{a^2}\int_S(\rho_s - 1)\mathbf{v}(\mathbf{r})e^{i\boldsymbol{\beta}\cdot\mathbf{r}}dS \\ &-i\boldsymbol{\beta}\cdot\overline{\mathbf{v}} = -i\omega B_0^{-1}\overline{p} - \frac{i\omega B_0^{-1}}{a^2}\int_S(B_s^{-1} - 1)p(\mathbf{r})e^{i\boldsymbol{\beta}\cdot\mathbf{r}}dS \\ &\text{where } \overline{\mathbf{v}} = \frac{1}{a^2}\int_S\mathbf{v}(\mathbf{r})e^{i\boldsymbol{\beta}\cdot\mathbf{r}}dS, \quad \text{and } \overline{p} = \frac{1}{a^2}\int_S p(\mathbf{r})e^{i\boldsymbol{\beta}\cdot\mathbf{r}}dS.\end{aligned} \tag{2}$$

Now, using Taylor's expansion in the long wavelength limit ($a << 2\pi/|\boldsymbol{\beta}|$),

$$\begin{aligned}&\int_S(\rho_s - 1)\mathbf{v}(\mathbf{r})e^{i\boldsymbol{\beta}\cdot\mathbf{r}}dS = \int_S(\rho_s - 1)\mathbf{v}(\mathbf{r})dS - i\boldsymbol{\beta}\times\int_S\frac{\mathbf{r}\times(\rho_s - 1)\mathbf{v}(\mathbf{r})}{2}dS + \cdots, \\ &\int_S(B_s^{-1} - 1)p(\mathbf{r})e^{i\boldsymbol{\beta}\cdot\mathbf{r}}dS = \int_S(B_s^{-1} - 1)p(\mathbf{r})dS - i\boldsymbol{\beta}\cdot\int_S\frac{\mathbf{r}(B_s^{-1} - 1)p(\mathbf{r})}{2}dS + \cdots\end{aligned} \tag{3}$$

(2) is rewritten as

$$
\begin{aligned}
&-i\boldsymbol{\beta}[\bar{p}-\frac{i\omega}{2a^2}\int_S \mathbf{r}\cdot(\rho_{sr}-1)\mathbf{v}(\mathbf{r})dS]=-i\omega\rho_0[\bar{\mathbf{v}}+\frac{1}{a^2}\int_S(\rho_s-1)\mathbf{v}(\mathbf{r})dS]\\
&-i\boldsymbol{\beta}\cdot[\bar{\mathbf{v}}-\frac{i\omega}{2a^2}\int_S \mathbf{r}(B_s^{-1}-1)p(\mathbf{r})dS]=-i\omega B_0^{-1}[\bar{p}+\frac{1}{a^2}\int_S(B_s^{-1}-1)p(\mathbf{r})dS],
\end{aligned}
\tag{4}
$$

where ρ_{sn} is ρ_s in the n (r or x) direction. Comparing the microscopic (1) and macroscopic (4) equations, we obtain the averaged fields p_{av}, $\boldsymbol{\pi}_{\text{av}}$, $\mathbf{v}_{\text{av}}$, and e_{av} as

$$
\begin{aligned}
&p_{\text{av}}=\bar{p}-\frac{i\omega}{2a^2}\int_S \mathbf{r}\cdot(\rho_{sr}-1)\mathbf{v}(\mathbf{r})dS, \qquad \boldsymbol{\pi}_{\text{av}}=\rho_0[\bar{\mathbf{v}}+\frac{1}{a^2}\int_S(\rho_s-1)\mathbf{v}(\mathbf{r})dS]\\
&\mathbf{v}_{\text{av}}=\bar{\mathbf{v}}-\frac{i\omega}{2a^2}\int_S \mathbf{r}(B_s^{-1}-1)p(\mathbf{r})dS, \qquad e_{\text{av}}=B_0^{-1}[\bar{p}+\frac{1}{a^2}\int_S(B_s^{-1}-1)p(\mathbf{r})dS],
\end{aligned}
\tag{5}
$$

which make the macroscopic acoustic equations (4) to take the usual forms of $-i\boldsymbol{\beta}p_{\text{av}}=-i\omega\boldsymbol{\pi}_{\text{av}}$, and $-i\boldsymbol{\beta}\cdot\mathbf{v}_{\text{av}}=-i\omega e_{\text{av}}$. Under good approximations of $\bar{v}_x\sim\frac{1}{a^2}\int_S v_x dS$ *and* $\bar{p}\sim\frac{1}{a^2}\int_S p dS,$ we finally obtain the effective parameters of ρ_x and B^{-1}:

$$
\begin{aligned}
&\rho_x=\frac{\pi_{\text{av},x}}{\rho_0 v_{\text{av},x}}=\frac{\bar{v}_x+\frac{1}{a^2}\int_S(\rho_{sx}-1)v_x dS}{\bar{v}_x-\frac{i\omega}{2a^2}\int_S(B_s^{-1}-1)px\,dS}\sim\frac{\int_S\rho_{sx}v_x dS}{\int_S v_x dS-\frac{i\omega}{2}\int_S(B_s^{-1}-1)px\,dS}, and\\
&B^{-1}=\frac{e_{\text{av}}}{B_0^{-1}p_{\text{av}}}=\frac{\bar{p}+\frac{1}{a^2}\int_S(B_s^{-1}-1)p\,dS}{\bar{p}+\frac{i\omega}{2a^2}\int_S r(\rho_{sr}-1)v_r dS}\sim\frac{\int_S B_s^{-1}p\,dS}{\int_S p\,dS+\frac{i\omega}{2}\int_S r(\rho_{sr}-1)v_r dS}.
\end{aligned}
\tag{6}
$$

Supplementary Note 2. Decoupling of the effective macroscopic acoustic parameters near the Dirac point

Near the Dirac point, the decoupling of ρ_x and B^{-1} can be achieved using a meta-atom having an inner sub-cell with radial symmetry and outer sub-cells supporting linear vibrations, constructed with a membrane, air, and solid walls (Fig. 1b in manuscript). Considering that the area (thickness) and compressibility of the membrane is much less than that of air such that $s_{\text{m}} \ll s_0$, $B_{s\text{m}}^{-1} \ll B_{s0}^{-1} = 1$, and noting that the mass of the membrane is much larger than that of air such that $\rho_{s\text{m}}s_{\text{m}} \gg \rho_{s0}s_0$, we achieve,

$$\rho_x \sim \frac{\int_{Sm} \rho_{mx} v_x dS}{\int_{S0} v_x dS + \frac{i\omega}{2}\int_{Sm} pxdS} = \frac{\int_{ISm} \rho_{mx} v_x dS + \int_{OSm} \rho_{mx} v_x dS}{\int_{IS0} v_x dS + \int_{OS0} v_x dS + \frac{i\omega}{2}\int_{Sm} pxdS}$$

$$B^{-1} \sim \frac{\int_{S0} B_{s0}^{-1} pdS}{\int_{S0} pdS + \frac{i\omega}{2}\int_{Sm} r\rho_{mr} v_r dS} = \frac{\int_{S0} B_{s0}^{-1} pdS}{\int_{S0} B_{s0}^{-1} pdS + \frac{i\omega}{2}(\int_{ISm} r\rho_{mr} v_r dS + \int_{OSm} r\rho_{mr} v_r dS)}. \quad (7)$$

where ISm(0) and OSm(0) refer to the integration region of the inner sub-cell and the outer sub-cell at the membrane (air), respectively. Now, near $B^{-1} \sim 0$ with zero effective compressibility, $\int_{OSm}(r\rho_{mr} v_r)dS \sim 0$ because the radial movement of the outer membrane is impossible, and because the first term in the denominator vanishes from $\int_{S0} pdS = B_{s0}{}^{-1}\int pdS \sim 0$ (with $B_{s0}{}^{-1} = 1$, and $B^{-1} \sim 0$ while B^{-1} proportional to $\int B_{s0}{}^{-1} pdS$), we obtain

$$B^{-1} = \frac{\int_{S0} B_{s0}^{-1} pdS}{\frac{i\omega}{2}\int_{ISm} r\rho_{mr} v_r dS}. \quad (8)$$

Similarly, near $\rho_x \sim 0$, the outer- and inner- membrane should move out of phase but need to maintain the same momentum value. To control ρ with the outer cells we impose $\int_{IS0} v_x dS \sim 0$, which can be achieved with a large ρ_m in the inner membrane because $\rho_x \sim \int_{ISm} \rho_{mx} v_x dS + \int_{OSm} \rho_{mx} v_x dS \sim 0$. Additionally, for the membrane, $s_m << s_0$ thus $\int_{Sm} pxdS << \int_{OS0} v_x dS$; thus,

$$\rho_x = \frac{\int_{ISm} \rho_{mx} v_x dS + \int_{OSm} \rho_{mx} v_x dS}{\int_{OS0} v_x dS}. \quad (9)$$

Supplementary Note 3. Coupled mode theory derivation of (ρ, B^{-1}) for a meta-atom having a sub-cell design

For a specific geometry of Fig. 1b in the manuscript, a more rigorous solution for (ρ, B^{-1}) can be obtained by solving the coupled mode equation. Assuming an average displacement $q_{[jk]}$ (i.e., membrane displacement directing from j to k) and pressure $p_{[i]}$ (i.e., pressure in the sub-cell room of i) as in Supplementary Fig. 1a, we separately apply Newton's law to the membranes and Hooke's

law to the sub-cells to obtain the relation between $p_{[i]}$ and $q_{[jk]}$. Assuming Floquet's boundary conditions with an acoustic refractive index of (n_x, n_y), the coupling equations are then expressed as

$$\begin{aligned} & B_0^{-1} a p_{[i]} s_{[i]} = \sum_k b_{[ik]} q_{[ik]} - \sum_j b_{[ji]} q_{[ji]}, && -\rho_{Al} t_{[jk]} \omega^2 q_{[jk]} = (p_{[k]} - p_{[j]}), \\ & \frac{p_{[7]} + p_{[3]}}{p_{[5]} + p_{[1]}} = \frac{q_{[37]}}{q_{[51]}} = \exp(-i\beta_0 n_x a), && \frac{p_{[2]} + p_{[6]}}{p_{[8]} + p_{[4]}} = \frac{q_{[26]}}{q_{[84]}} = \exp(-i\beta_0 n_y a) \end{aligned} \tag{10}$$

where $b_{[jk]}$, $t_{[jk]}$ and $s_{[i]}$ are the width, thickness and area of the membrane $[jk]$ and sub-cell $[i]$; the B_0, β_0 are the air modulus and propagation constant, ρ_{Al} is the Aluminum density and a is the lattice constant.

To find the eigenmodes of the system, we rewrite (10) in the form of a linear system (**Ax** = **0**), where **A** is the 17 x 17 matrix, and **x** = [***p***, ***q***] is the vector consisting of p's and q's. Focusing on the x direction ($n_x = n$, $n_y = 0$), (10) can be written as

$$\begin{pmatrix}
1 & 0 & 0 & 0 & 0 & 0 & 0 & 0 & 0 & C_{\text{O}} & 0 & 0 & 0 & -C_{\text{O}} & 0 & 0 & 0 \\
0 & 1 & 0 & 0 & 0 & 0 & 0 & 0 & 0 & 0 & -C_{\text{O}} & 0 & 0 & 0 & C_{\text{O}} & 0 & 0 \\
0 & 0 & 1 & 0 & 0 & 0 & 0 & 0 & 0 & 0 & 0 & -C_{\text{O}} & 0 & 0 & 0 & C_{\text{O}} & 0 \\
0 & 0 & 0 & 1 & 0 & 0 & 0 & 0 & 0 & 0 & 0 & 0 & C_{\text{O}} & 0 & 0 & 0 & -C_{\text{O}} \\
0 & 0 & 0 & 0 & 0 & 0 & 0 & 0 & 1 & -C_{\text{I}} & C_{\text{I}} & C_{\text{I}} & -C_{\text{I}} & 0 & 0 & 0 & 0 \\
1 & 0 & 0 & 0 & -1 & 0 & 0 & 0 & 0 & 0 & 0 & 0 & 0 & M_{\text{O}} & 0 & 0 & 0 \\
0 & 1 & 0 & 0 & 0 & -1 & 0 & 0 & 0 & 0 & 0 & 0 & 0 & 0 & -M_{\text{O}} & 0 & 0 \\
0 & 0 & 1 & 0 & 0 & 0 & -1 & 0 & 0 & 0 & 0 & 0 & 0 & 0 & 0 & -M_{\text{O}} & 0 \\
0 & 0 & 0 & 1 & 0 & 0 & 0 & -1 & 0 & 0 & 0 & 0 & 0 & 0 & 0 & 0 & M_{\text{O}} \\
1 & 0 & 0 & 0 & 0 & 0 & 0 & 0 & -1 & -M_{\text{I}} & 0 & 0 & 0 & 0 & 0 & 0 & 0 \\
0 & 1 & 0 & 0 & 0 & 0 & 0 & 0 & -1 & 0 & M_{\text{I}} & 0 & 0 & 0 & 0 & 0 & 0 \\
0 & 0 & 1 & 0 & 0 & 0 & 0 & 0 & -1 & 0 & 0 & M_{\text{I}} & 0 & 0 & 0 & 0 & 0 \\
0 & 0 & 0 & 1 & 0 & 0 & 0 & 0 & -1 & 0 & 0 & 0 & -M_{\text{I}} & 0 & 0 & 0 & 0 \\
0 & 0 & 0 & 0 & 0 & 0 & 0 & 0 & 0 & 0 & 0 & 0 & 0 & e^{-i\beta_0 na} & 0 & -1 & 0 \\
0 & 0 & 0 & 0 & 0 & 0 & 0 & 0 & 0 & 0 & 0 & 0 & 0 & 0 & 1 & 0 & -1 \\
e^{-i\beta_0 na} & 0 & -1 & 0 & e^{-i\beta_0 na} & 0 & -1 & 0 & 0 & 0 & 0 & 0 & 0 & 0 & 0 & 0 & 0 \\
0 & 1 & 0 & -1 & 0 & 1 & 0 & -1 & 0 & 0 & 0 & 0 & 0 & 0 & 0 & 0 & 0
\end{pmatrix}
\begin{pmatrix} p_{[1]} \\ p_{[2]} \\ p_{[3]} \\ p_{[4]} \\ p_{[5]} \\ p_{[6]} \\ p_{[7]} \\ p_{[8]} \\ p_{[9]} \\ q'_{[19]} \\ q'_{[92]} \\ q'_{[93]} \\ q'_{[49]} \\ q'_{[51]} \\ q'_{[26]} \\ q'_{[37]} \\ q'_{[84]} \end{pmatrix}
=
\begin{pmatrix} 0 \\ 0 \\ 0 \\ 0 \\ 0 \\ 0 \\ 0 \\ 0 \\ 0 \\ 0 \\ 0 \\ 0 \\ 0 \\ 0 \\ 0 \\ 0 \\ 0 \end{pmatrix} \tag{11}$$

where $C_{\text{I}} = \frac{B_0 a}{s_{\text{I}}}, C_{\text{O}} = \frac{B_0 a}{s_{\text{O}}}, M_{\text{I}} = -\frac{\omega^2 a}{a_{\text{in}}}[\rho_{\text{Al}} t_{\text{I}} + \frac{\rho_0 s_{\text{O}}}{2a_{\text{in}}} + \frac{\rho_0 s_{\text{I}}}{4a_{\text{in}}}], M_{\text{O}} = -2\omega^2[\rho_{\text{Al}} t_{\text{O}} + \frac{\rho_0 s_{\text{O}}}{2a}]$ and $q'_n = \frac{b_n}{a_n} q_n$.

It should be noted that the matrix **A** in (11) consists of the coefficients from Hooke's law (row 1 to 5), Newton's law (row 6 to 13), and Floquet's boundaries (row 14 to 17).

To achieve a non-zero physical null space of $\mathbf{x} = [\boldsymbol{p}, \boldsymbol{q}]$, Det(**A**) should vanish, leading to the *analytical expression for effective n*:

$$\exp(i\beta_0 na) = \frac{1}{2C_\mathrm{O}^2 C_\mathrm{I}}\{M_\mathrm{O}(M_\mathrm{I}+C_\mathrm{O})(M_\mathrm{I}+C_\mathrm{O}+4C_\mathrm{I}) + 2C_\mathrm{O}[M_\mathrm{I}(M_\mathrm{I}+C_\mathrm{O}) + C_\mathrm{I}(4M_\mathrm{I}+C_\mathrm{O})] \pm \sqrt{[2M_\mathrm{I}C_\mathrm{O} + M_\mathrm{O}(M_\mathrm{I}+C_\mathrm{O})][M_\mathrm{I}+C_\mathrm{O}+4C_\mathrm{I}][2M_\mathrm{I}C_\mathrm{O}(M_\mathrm{I}+C_\mathrm{O}) + 4C_\mathrm{O}C_\mathrm{I}(2M_\mathrm{I}+C_\mathrm{O}) + M_\mathrm{O}(M_\mathrm{I}+C_\mathrm{O})(M_\mathrm{I}+C_\mathrm{O}+4C_\mathrm{I})]}\}. \tag{12}$$

The effective impedance Z then also becomes

$$ZZ_0 = \frac{2i\omega q'_{[7]}}{p_{[7]} + p_{[3]}} = \pm\frac{2i\omega(M_\mathrm{I}+C_\mathrm{O})\sqrt{M_\mathrm{I}+C_\mathrm{O}+4C_\mathrm{I}}}{\sqrt{[2M_\mathrm{I}C_\mathrm{O} + M_\mathrm{O}(M_\mathrm{I}+C_\mathrm{O})][2M_\mathrm{I}C_\mathrm{O}(M_\mathrm{I}+C_\mathrm{O}) + 4C_\mathrm{O}C_\mathrm{I}(2M_\mathrm{I}+C_\mathrm{O}) + M_\mathrm{O}(M_\mathrm{I}+C_\mathrm{O})(M_\mathrm{I}+C_\mathrm{O}+4C_\mathrm{I})]}}. \tag{13}$$

Using $\rho = n/Z$ and $B^{-1} = nZ$, we finally obtain rigorous solutions for ρ and B in analytical forms.

Of critical importance is in solving the inverse problem, to find the design parameters of the membranes (M_O, M_I) from the target wave parameters of n and Z. From (12) and (13), we obtain,

$$\begin{aligned} M_\mathrm{O} &= -2C_\mathrm{O} \pm_1 \frac{2\omega[1+\cos(\frac{\omega na}{c_0})]}{Z_0 Z\sin(\frac{\omega na}{c_0})} \pm_2 \frac{4C_\mathrm{I}\omega\sqrt{[1\pm_1 \frac{C_\mathrm{O}^2}{4C_\mathrm{I}\omega}Z_0 Z\sin(\frac{\omega na}{c_0})]}}{C_\mathrm{I}Z_0 Z\sin(\frac{\omega na}{c_0})} \\ M_\mathrm{I} &= -C_\mathrm{O} - 2C_\mathrm{I} + (\pm_1\cdot\pm_2)\frac{2C_\mathrm{I}\omega\sqrt{[1\pm_1 \frac{C_\mathrm{O}^2}{4C_\mathrm{I}\omega}Z_0 Z\sin(\frac{\omega na}{c_0})]}}{\omega}. \end{aligned} \tag{14}$$

Simplifying (14) in the long wavelength limit [$\exp(i\beta_0 na) \sim 1 + i\beta_0 na - 1/2(\beta_0 na)^2$] and by setting $\pm_1 = +$ and $\pm_2 = -$ for the condition of (Im[n] ≥ 0, Re[Z] ≥ 0), for a first order approximation we achieve decoupled equations for M_O (ρ) and M_I (B^{-1}) that is equivalent to equation (3) in the manuscript:

$$\begin{aligned} -(t_\mathrm{O}\rho_\mathrm{Al} + \frac{\rho_0 s_\mathrm{O}}{2a})\omega^2 &= \frac{1}{2}M_\mathrm{O} \sim -C_\mathrm{O} + \frac{\omega[2-\frac{1}{2}(\frac{\omega na}{c_0})^2]}{Z_0 Z\frac{\omega na}{c_0}} - \frac{2\omega[1+\frac{C_\mathrm{O}^2}{8C_\mathrm{I}\omega}Z_0 Z\frac{\omega na}{c_0}]}{Z_0 Z\frac{\omega na}{c_0}} = -C_\mathrm{O} - \frac{1}{2}\omega^2 a\rho\rho_0 - \frac{C_\mathrm{O}^2}{4C_\mathrm{I}} = -a(\frac{B_0}{s_\mathrm{O}} + \frac{B_0 s_\mathrm{I}}{4s_\mathrm{O}^2} + \frac{1}{2}\omega^2\rho\rho_0) \\ -(t_\mathrm{I}\rho_\mathrm{Al} + \frac{\rho_0 s_\mathrm{O}}{2a_\mathrm{in}} + \frac{\rho_0 s_\mathrm{I}}{4a_\mathrm{in}})\omega^2 a/a_\mathrm{in} &= M_\mathrm{I} \sim -C_\mathrm{O} - 2C_\mathrm{I} - 2C_\mathrm{I}[1+\frac{C_\mathrm{O}^2}{8C_\mathrm{I}}(\frac{Z_0 Zna}{c_0})] = -C_\mathrm{O} - 4C_\mathrm{I} - \frac{C_\mathrm{O}^2 a}{4}\frac{B^{-1}}{B_0^{-1}} = -a(\frac{B_0}{s_\mathrm{O}} + \frac{4B_0}{s_\mathrm{I}} + (\frac{B_0 a^2}{4s_\mathrm{O}^2}\frac{B^{-1}}{B_0^{-1}})). \end{aligned} \tag{15}$$

From the CMT (11), the dispersion relation $\omega(k)$ also can be obtained. Replacing M_I and M_O with $m_\mathrm{I}\omega^2$ and $m_\mathrm{O}\omega^2$ respectively, the CMT now becomes the function of ω and k. Directly solving Det[**A**]=0 in terms of wavenumber $\omega(k)$, we obtain,

$$
\begin{aligned}
\omega = & \; 0,\ \pm i\frac{\sqrt{C_\mathrm{O}}}{\sqrt{m_\mathrm{I}}},\ \pm\frac{\sqrt{-(m_\mathrm{I}+m_\mathrm{O})C_\mathrm{O}}}{\sqrt{m_\mathrm{I}}\sqrt{m_\mathrm{O}}},\\
& \pm\sqrt{\left[-\frac{2C_\mathrm{O}}{m_\mathrm{O}}-\frac{2C_\mathrm{O}}{m_\mathrm{I}}-\frac{4C_\mathrm{I}}{m_\mathrm{I}}+\frac{2^{-1/3}}{m_\mathrm{O}m_\mathrm{I}}P+\frac{2^{1/3}}{m_\mathrm{I}}\frac{Q}{P}\right]/3},\\
& \pm\sqrt{\left[-\frac{2C_\mathrm{O}}{m_\mathrm{O}}-\frac{2C_\mathrm{O}}{m_\mathrm{I}}-\frac{4C_\mathrm{I}}{m_\mathrm{I}}+\frac{2^{-1/3}}{m_\mathrm{O}m_\mathrm{I}}\left(\frac{-1-\sqrt{3}i}{2}\right)P+\frac{2^{1/3}}{m_\mathrm{I}}\left(\frac{-1+\sqrt{3}i}{2}\right)\frac{Q}{P}\right]/3},\\
& \pm\sqrt{\left[-\frac{2C_\mathrm{O}}{m_\mathrm{O}}-\frac{2C_\mathrm{O}}{m_\mathrm{I}}-\frac{4C_\mathrm{I}}{m_\mathrm{I}}+\frac{2^{-1/3}}{m_\mathrm{O}m_\mathrm{I}}\left(\frac{-1+\sqrt{3}i}{2}\right)P+\frac{2^{1/3}}{m_\mathrm{I}}\left(\frac{-1-\sqrt{3}i}{2}\right)\frac{Q}{P}\right]/3}
\end{aligned}
$$

$$
\mathit{where}\left(\begin{aligned}
&\Pi = 2\left(m_\mathrm{O}{}^3+3m_\mathrm{O}{}^2m_\mathrm{I}-6m_\mathrm{O}m_\mathrm{I}{}^2-8m_\mathrm{I}{}^3\right)C_\mathrm{O}{}^3+12\left(m_\mathrm{O}{}^3-m_\mathrm{O}{}^2m_\mathrm{I}+4m_\mathrm{O}m_\mathrm{I}{}^2\right)C_\mathrm{O}{}^2C_\mathrm{I}\\
&\qquad +54m_\mathrm{O}{}^2m_\mathrm{I}\left[\cosh(k_xa)+\cosh(k_ya)\right]C_\mathrm{O}{}^2C_\mathrm{I}+48\left(-m_\mathrm{O}{}^3+2m_\mathrm{O}{}^2m_\mathrm{I}\right)C_\mathrm{O}C_\mathrm{I}^2-128m_\mathrm{O}{}^3C_\mathrm{I}^3\\
&P = \left[\Pi+\sqrt{\Pi^2-4\left[\left(m_\mathrm{O}{}^2+2m_\mathrm{O}m_\mathrm{I}+4m_\mathrm{I}{}^2\right)C_\mathrm{O}{}^2+4\left(m_\mathrm{O}{}^2-2m_\mathrm{O}m_\mathrm{I}\right)C_\mathrm{O}C_\mathrm{I}+16m_\mathrm{O}{}^2C_\mathrm{I}{}^2\right]^3}\right]^{\frac{1}{3}}\\
&Q = \left(m_\mathrm{O}+2m_\mathrm{I}+4m_\mathrm{I}{}^2/m_\mathrm{O}\right)C_\mathrm{O}{}^2+4\left(m_\mathrm{O}-2m_\mathrm{I}\right)C_\mathrm{O}C_\mathrm{I}+16m_\mathrm{O}C_\mathrm{I}{}^2
\end{aligned}\right) \tag{16}
$$

Excluding negative valued and trivial solutions, we get solutions corresponding to 5 lowest bands; two flat bands and three dispersive bands (Supplementary Fig. 2a). Focusing on the Dirac point[6,7] (ρ, B^{-1}) = (0,0), we plot band diagram near 1,300Hz as shown in Supplementary Fig. 2b; exhibiting the Dirac cone and a third flat band (corresponding to the acoustic transverse mode[6]) providing a triple degeneracy at $k = 0$.

Supplementary Note 4. Characteristic motions of dipolar (ρ) and monopolar (B^{-1}) eigenmodes at the Dirac point

Combining the eigensolution sets of **v** and p that were calculated in Supplementary Note 3 meanwhile considering the excitation directions, it is possible to extract the dipolar-ρ (linear vibrations) and monopolar-B^{-1} (radial vibrations) modes, of the proposed structure. In Supplementary Fig. 3, we plot the CMT-calculated eigenmode patterns and displacement of membranes (gray lines) near the Dirac point, which clearly shows negligible vibrations of the inner membranes for the ρ - dipolar mode and negligible vibrations of the outer membrane for the B^{-1} - monopolar mode. Black arrows indicate the momentum of the individual membranes. These results confirm the proposed ansatzs and discussions regarding Eq. (2) in the manuscript.

Supplementary Note 5. Decoupled operation away from the Dirac point and Extension of the tuning range

To shift the center of decoupling operation away from the Dirac point (ρ, B^{-1}) = (0, 0), we place the meta-atom in the host medium of (ρ_h, B_h^{-1}) (Supplementary Fig. 4a). In the frame of the effective medium theory, the shifted center of decoupling operation is calculated from (ρ_c, B_c^{-1}) = (ρ_h (1-a/a_h), B_h^{-1}(1-a^2/a_h^2)), well agreeing with the FEM results (Supplementary Fig. 4b-d). For example, with air (ρ_h, B_h^{-1}) = (1, 1) and a_h = 7.5cm, it was possible to shift the decoupled operation point to (ρ_c, B_c^{-1}) = (0.2, 0.36); mixing with (ρ_h, B_h^{-1}) = (2, 2) we also get (0.4, 0.72).

For the extension of effective parameters' tuning ranges, we first focus on the extension of ρ, with Eq. (3) in the manuscript and (15). With $\rho \sim \rho_{Al} t_O / a$ and $B^{-1} \sim \rho_{Al} t_I\, a$ (as $s_O \sim a^2$), the tuning range of ρ is extended (while keeping B^{-1} at same value) by increasing t_O and t_I, and using smaller unit cell a. For example, we get ρ = -36~36 and B^{-1} = -1~1 with a = 1.0cm & thicker t_O and t_I (Supplementary Fig. 5), as intuitively expected for the smaller and heavier meta-atom. The same equation (3) in the manuscript and (15) can be used to change the tuning range of B^{-1}. Nonetheless, it is noted that the tuning range of B^{-1} = -1~1 is already not small, because B^{-1} is normalized to Air (B_{Air}^{-1} : $B_{Solid\ or\ Liquid}^{-1}$ = $10^4 \sim 10^6$: 1)[8].

Supplementary Note 6. Coupled mode theory derivation of bianisotropy ξ for a meta-atom having a asymmetric sub-cell design

Decoupled control over the bianisotropy ξ [9-11] of the acoustic meta-atom also can be achieved with the asymmetric assignment of the inner membrane thickness (non-zero $\Delta t_I = t_{I\text{-}Right} - t_{I\text{-}Left}$), as shown in Supplementary Fig. 6. The analytical formula for ξ is obtained by solving the coupled mode equations using a similar process to that described in Supplementary Note 3. Solving for Z_+ and Z_- for $+x$ and $-x$ propagating waves, respectively, $\xi = in(Z_+ - Z_-)/(Z_+ + Z_-)$ becomes

$$\xi \sim -2\frac{c_0 C_O^{\ 2}}{a\omega}(M_I + C_O + 2C_I)\Delta M \frac{1}{F}\log\left(\frac{1}{(2C_O^2(M_I + C_O)C_I)(G-F)}\right) \tag{17}$$

$$\text{where}\begin{cases} F=\sqrt{(M_{\text{I}}+C_{\text{O}})(H-K+2C_{\text{I}}(2H-K+2C_{\text{I}}(2H-K)]} \\ G=(M_{\text{I}}+C_{\text{O}})(H-K)+2C_{\text{I}}(2H-K+2M_{\text{I}}C_{\text{O}}+C_{\text{O}}^2) \\ H=(M_{\text{I}}+C_{\text{O}})(2M_{\text{I}}C_{\text{O}}+M_{\text{O}}(M_{\text{I}}+C_{\text{O}})) \\ K=(M_{\text{O}}+2C_{\text{O}})\Delta M^2 \end{cases} \text{and}\,\Delta M=-\frac{a\omega^2\rho_{\text{Al}}}{a_{\text{in}}}\Delta t_{\text{I}}.$$

In a good approximation to the first order Δt_{I} near the Dirac point ($\rho = B^{-1} = 0$), the bianisotropy ξ of the meta-atom is solely determined by the structural asymmetry Δt_{I}, as shown below, which is independent (completely decoupled) from t_{I} or t_{O}:

$$\begin{aligned}\xi &= \frac{4c_0C_{\text{I}}}{a\omega\sqrt{\Delta M^2+16C_{\text{I}}^2}}\log(1+\frac{\Delta M^2}{8C_{\text{I}}^2}+\frac{\Delta M\sqrt{\Delta M^2+16C_{\text{I}}^2}}{C_{\text{I}}^2}) \\ &\sim \frac{c_0}{2aC_{\text{I}}\omega}\Delta M = -\frac{c_0s_{\text{I}}\omega\rho_{\text{Al}}}{B_02aa_{\text{in}}}\Delta t_{\text{I}}.\end{aligned} \tag{18}$$

Supplementary Note 7. Condition of complete tunneling between asymmetric impedance waveguides with bianisotropic meta-waveguide

In this section, we consider the problem of perfect tunneling between two different impedance waveguides (region I and region III, $Z_{\text{I}} \neq Z_{\text{III}}$), with the introduction of bianisotropic media (ρ, B^{-1}, ξ) in their interface (region II, Supplementary Fig. 7, left). Assuming the incident wave from the left, we convert the well-known electromagnetic wave equations of bianisotropy[9] into the acoustic wave equations; by replacing E and H with v and p, and applying the boundary conditions we get,

$$\begin{cases} v_{\text{II}} = v_{\text{II}+}e^{-ik_0nx}+v_{\text{II}-}e^{ik_0nx} \\ p_{\text{II}} = \frac{v_{\text{II}+}}{Z_+}e^{-ik_0nx}-\frac{v_{\text{II}-}}{Z_-}e^{ik_0nx} \end{cases}, \quad v_{\text{II}}(0)=1+R, v_{\text{II}}(d)=\sqrt{\frac{Z_{\text{III}}}{Z_{\text{I}}}}T, p_{\text{II}}(0)=\frac{1-R}{Z_{\text{I}}}, p_{\text{II}}(d)=\sqrt{\frac{1}{Z_{\text{I}}Z_{\text{III}}}}T \tag{19}$$

where n is the refractive index; Z_+ and Z_- are the impedances for the + and - directions ($Z_+ \neq Z_-$ when $\xi \neq 0$), respectively; R and T denote the reflection and transmission coefficients across region II. To achieve 100% transmittance with zero phase shift, we impose $R = 0$ and $T = 1$ to obtain

$$\begin{cases} v_{\mathrm{II}+} + v_{\mathrm{II}-} = 1 \\ v_{\mathrm{II}+} e^{-ik_0 nd} + v_{\mathrm{II}-} e^{ik_0 nd} = \sqrt{\dfrac{Z_{\mathrm{III}}}{Z_{\mathrm{I}}}} \\ \dfrac{v_{\mathrm{II}+}}{Z_+} - \dfrac{v_{\mathrm{II}-}}{Z_-} = \dfrac{1}{Z_{\mathrm{I}}} \\ \dfrac{v_{\mathrm{II}+}}{Z_+} e^{-ik_0 nd} - \dfrac{v_{\mathrm{II}-}}{Z_-} e^{ik_0 nd} = \sqrt{\dfrac{1}{Z_{\mathrm{I}} Z_{\mathrm{III}}}} \end{cases} \tag{20}$$

Eliminating $v_{\mathrm{II}+}$ and $v_{\mathrm{II}-}$ in (20), we get

$$\begin{cases} \dfrac{Z_+(Z_{\mathrm{I}} + Z_-)}{Z_{\mathrm{I}}(Z_+ + Z_-)} e^{-ik_0 nd} + \dfrac{Z_-(Z_{\mathrm{I}} - Z_+)}{Z_{\mathrm{I}}(Z_+ + Z_-)} e^{ik_0 nd} = \sqrt{\dfrac{Z_{\mathrm{III}}}{Z_{\mathrm{I}}}} \\ \dfrac{Z_{\mathrm{I}} + Z_-}{Z_{\mathrm{I}}(Z_+ + Z_-)} e^{-ik_0 nd} - \dfrac{Z_{\mathrm{I}} - Z_+}{Z_{\mathrm{I}}(Z_+ + Z_-)} e^{ik_0 nd} = \sqrt{\dfrac{1}{Z_{\mathrm{I}} Z_{\mathrm{III}}}} \end{cases} \tag{21}$$

Additional equations can be obtained from the other case, where the wave propagates from the region of input impedance Z_{III} to the region of the output impedance Z_{I}. By exchanging Z_{I} and Z_{III} and Z_+ and Z_- in (21), we get two additional equations, and together with (21) we achieve the following expressions for $\exp(ik_0 nd)$:

$$e^{ik_0 nd} = \sqrt{\frac{Z_{\mathrm{I}}}{Z_{\mathrm{III}}}} \frac{Z_{\mathrm{III}} - Z_+}{Z_{\mathrm{I}} - Z_+} = \sqrt{\frac{Z_{\mathrm{I}}}{Z_{\mathrm{III}}}} \frac{Z_{\mathrm{III}} + Z_+}{Z_{\mathrm{I}} + Z_+} = \sqrt{\frac{Z_{\mathrm{III}}}{Z_{\mathrm{I}}}} \frac{Z_{\mathrm{I}} - Z_-}{Z_{\mathrm{III}} - Z_-} = \sqrt{\frac{Z_{\mathrm{III}}}{Z_{\mathrm{I}}}} \frac{Z_{\mathrm{I}} + Z_-}{Z_{\mathrm{III}} + Z_-}. \tag{22}$$

It is evident that (22) holds only if $Z_+ = 0$ and $Z_- = \infty$ (or if $Z_+ = \infty$ and $Z_- = 0$). This leads to two sets of solutions $(n, Z_+, Z_-) = (1/2\log(Z_{\mathrm{III}}/Z_{\mathrm{I}})/ik_0 d, 0, \infty)$ and $(1/2\log(Z_{\mathrm{I}}/Z_{\mathrm{III}})/ik_0 d, \infty, 0)$, which are in fact physically identical in terms of $(\rho, B^{-1}, \xi) = (2n/(Z_+ + Z_-), 2Z_+ Z_- n/(Z_+ + Z_-), (Z_+ - Z_-)/(Z_+ + Z_-)) = (0, 0, 1/2\log(Z_{\mathrm{I}}/Z_{\mathrm{III}})/k_0 d)$. It is important to note that when the impedances of the input and output waveguides are identical ($Z_{\mathrm{I}} = Z_{\mathrm{III}}$), all solutions of ρ, B^{-1} and ξ should be zero concurrently; enforcing region II to be matched zero index material (for the initial condition of $R = 0$ and $T = 1$). In contrast, when $Z_{\mathrm{I}} \neq Z_{\mathrm{III}}$, the required bianisotropy $|\xi|$ increases for larger impedance mismatch $Z_{\mathrm{I}}/Z_{\mathrm{III}}$.

The condition for complete transmission between different width waveguides ($w_1 \neq w_2$, right of Supplementary Fig. 7) is also obtained by replacing Z with w, which leads to the solution $(\rho, B^{-1}, \xi) = (0, 0, 1/2\log(w_1/w_2)/k_0 d)$. It is noted that the proposed bianisotropic impedance conversion is intrinsically different from the resonator-based impedance matching[12], as our condition is determined only by the ratio of impedances ($Z_{\mathrm{III}}/Z_{\mathrm{I}}$), meanwhile the resonator approach involves both the matching of impedances $(Z_{\mathrm{I}} Z_{\mathrm{III}})^{1/2}$ and resonance $n_{\mathrm{eff}} \cdot d = \lambda/4$.

Supplementary Note 8. Transmittance of bianisotropic impedance conversion as a function of ξ

In this section, we calculate the transmittance for the problem shown in Supplementary Fig. 7 when the wave parameters of the medium in region II are (ρ, B^{-1}, ξ) = (0, 0, ξ). For this case, the wave equations in (19) are reduced to

$$\begin{cases} v_{\mathrm{II}} = v_{\mathrm{II}+}e^{k_0\xi x} \\ p_{\mathrm{II}} = p_{\mathrm{II}-}e^{-k_0\xi x} \end{cases}, \quad v_{\mathrm{II}}(0) = 1+R, v_{\mathrm{II}}(d) = \sqrt{\frac{Z_{\mathrm{III}}}{Z_{\mathrm{I}}}}T, \quad p_{\mathrm{II}}(0) = \frac{1-R}{Z_{\mathrm{I}}}, p_{\mathrm{II}}(d) = \sqrt{\frac{1}{Z_{\mathrm{I}}Z_{\mathrm{III}}}}T. \tag{23}$$

Solving (23) and also replacing Z with w, the transmittance T as a function of ξ is derived as

$$T = \frac{2}{\sqrt{\frac{Z_{\mathrm{III}}}{Z_{\mathrm{I}}}}e^{-k_0\xi d} + \sqrt{\frac{Z_{\mathrm{I}}}{Z_{\mathrm{III}}}}e^{k_0\xi d}} = \frac{2}{\sqrt{\frac{w_2}{w_1}}e^{-k_0\xi d} + \sqrt{\frac{w_1}{w_2}}e^{k_0\xi d}}. \tag{24}$$

From (24), the maximum transmittance (T = 1) is shown to occur when $\exp(2k_0\xi d) = Z_{\mathrm{III}}/Z_{\mathrm{I}}$, which is equivalent to the primary result obtained in Supplementary Note 7.

Supplementary Note 9. Determination of ρ, B^{-1} and ξ for the target ϕ_R, ϕ_T

With the target S_{11} (= $\sqrt{1/2}\exp(i\phi_R)$), S_{21} (= $\sqrt{1/2}\exp(i\phi_T)$), and S_{22} (= $\sqrt{1/2}\exp(i\phi)$) for the bianisotropic meta-atom (Supplementary Fig. 8), it is straightforward to calculate ρ, B^{-1} and ξ. Adapted from the corresponding solutions in electromagnetic waves[9] we have,

$$n = \frac{\cos^{-1}\left(\frac{1-S_{11}S_{22}+S_{21}^2}{2S_{21}}\right)}{k_0 d}, \quad \xi = \frac{n\frac{S_{11}-S_{22}}{S_{21}}}{-2\sin(nk_0 d)}, \quad B^{-1} = \frac{-in}{\sin(nk_0 d)}\left[\frac{1+S_{11}+S_{22}+S_{11}S_{22}-S_{21}^2}{2S_{21}}\right], \quad \rho = \frac{n^2+\xi^2}{B^{-1}}. \tag{25}$$

Assuming the lossless case (i.e., n is real or pure imaginary), we obtain real-valued ξ and B^{-1} if $\mathrm{Im}[(S_{11}-S_{22})/S_{21}] = 0$ and $\mathrm{Re}[(1+S_{11}+S_{22}+S_{11}S_{22}-S_{21}^2)/2S_{21}] = 0$. Then we get,

$$\mathrm{Im}(\frac{S_{11}-S_{22}}{S_{21}}) = \mathrm{Im}(\frac{e^{i\phi_\mathrm{R}}-e^{i\phi}}{e^{i\phi_\mathrm{T}}}) = \sin(\phi_\mathrm{R}-\phi_\mathrm{T}) - \sin(\phi-\phi_\mathrm{T}) = 2\sin(\frac{\phi_\mathrm{R}-\phi}{2})\cos(\frac{\phi+\phi_\mathrm{R}-2\phi_\mathrm{T}}{2}) = 0$$

$$\mathrm{Re}(\frac{1+S_{11}+S_{22}+S_{11}S_{22}-S_{21}^2}{2S_{21}}) = \frac{1}{2\sqrt{2}}[\cos(\phi_\mathrm{T})+\cos(\phi+\phi_\mathrm{R}-\phi_\mathrm{T})] + \frac{1}{2}[\cos(\phi_\mathrm{R}-\phi_\mathrm{T})+\cos(\phi-\phi_\mathrm{T})] \tag{26}$$

$$= \cos(\frac{\phi+\phi_\mathrm{R}-2\phi_\mathrm{T}}{2})[\cos(\frac{\phi_\mathrm{R}-\phi}{2}) + \frac{1}{\sqrt{2}}\cos(\frac{\phi_\mathrm{R}+\phi}{2})] = 0.$$

Both equations in (26) are satisfied when $\phi = \pi - \phi_\mathrm{R} + 2\phi_\mathrm{T}$. Using the target values ϕ_R, ϕ_T, and $\phi = \pi - \phi_\mathrm{R} + 2\phi_\mathrm{T}$ to calculate S_{11}, S_{21}, and S_{22}, then the required values of (ρ, B^{-1}, ξ) are determined using (25). Figure 4a in the manuscript and Supplementary Fig. 9 show the phase shift contour (ϕ_R, ϕ_T) in the parameter octant space of (ρ, B^{-1}, ξ) and the maps of each required wave parameters for the decoupled manipulation of the phase shift for (ϕ_R, ϕ_T) subject to the constraint of 50:50 power division. For example, Supplementary Fig. 10 shows the required phase shifts (ϕ_R, ϕ_T) of an individual meta-atom (in a single sheet of 40 × 1 array) achieving ordinary ($\Delta\phi(x) = 0$, blue marks) or extra-ordinary ($\Delta\phi(x) \neq 0$, red marks) reflection and transmission. From the target (ϕ_R, ϕ_T) values in Supplementary Fig. 10, the calculation of (ρ, B^{-1}, ξ) are obtained from Eqs. (25); then, the top-down determination of the corresponding (t_O, t_I, Δt_I) is straightforward.

Supplementary References

1. Cummer, S. A. & Schurig, D. One Path to acoustic cloaking. *New J. of Phys.* **9**, 45 (2007).
2. Timoshenko, S. & Woinowsky-Krieger, S. *Theory of plates and shells* (McGraw-Hill, New York, 1989).
3. Alù, A. First-principles homogenization theory for periodic metamaterials. *Phys. Rev. B* **84**, 075153 (2011).
4. Smith, D. R., Vier, D. C., Koschny, T. & Soukoulis, C. M. Electromagnetic parameter retrieval from inhomogeneous metamaterials. *Phys. Rev. E* **71**, 036617 (2005).
5. Silveirinha, M. G. Metamaterial homogenization approach with application to the characterization of microstructured composites with negative parameters. *Phys. Rev. B* **75**, 115104 (2007).

6. Liu, F., Huang, X. & Chan, C. T. Dirac cones at k = 0 in acoustic crystals and zero refractive index acoustic materials. *Appl. Phys. Lett.* **100**, 071911 (2012).

7. Huang X. *et al*. Dirac cones induced by accidental degeneracy in photonic crystals and zero-refractive-index materials. *Nat. Materials* **10**, 582-586 (2011).

8. Kinsler, L. E., Frey, A. R., Coppens, A. B., and Sanders, J. V. *Fundamental of acoustics* (John Wiley & Sons, 1999, USA).

9. Li., Z., Aydin, K. & Ozbay, E. Determination of the effective constitutive parameters of bianisotropic metamaterials from reflection and transmission coefficients. *Phys Rev. E* **79**, 026610 (2009).

10. Saadoun, M. M. I. & Engheta, N. A reciprocal phase shifter using novel pseudochiral or ω medium. *Microw. Opt. Technol. Lett.* **5**, 184-188 (1992).

11. Mackay, T. G. & Lakhtakia, A. *Electromagnetic anisotropy and bianisotropy: A field guide* (World Scienctific, Singapore, 2010).

12. Cheng, D. K. *Field and wave electromagnetics* (Addison Wesley, USA, 1989).
